# Induction of Labour in Advanced Abdominal Pregnancy with Fetal Demise due to Cord Round Neck: A Case Report of a Missed Diagnosis

**DOI:** 10.1155/2018/4171604

**Published:** 2018-09-25

**Authors:** Ascensius Achuo Mforteh, Collince Tchakounte, Carlson-Babila Sama, Stephane Eteme-Messi, Willy Djiognouo, Sama Dohbit, Pascal Foumane

**Affiliations:** ^1^Department of Obstetrics and Gynecology, University of Yaoundé 1, Yaoundé, Cameroon; ^2^Hopital Saint Jean de Malte, Njombe, Cameroon; ^3^Institute of Global Health, University College London, London, UK

## Abstract

**Background:**

There are increasing reports of term live abdominal pregnancies even though the diagnosis of abdominal pregnancy is made preoperatively only in 45% of cases which partly explains the high maternal and perinatal morbidity and mortality associated with abdominal pregnancy.

**Case Report:**

We report a rare case of misdiagnosed term abdominal pregnancy complicated by fetal demise due to cord round neck in a 29-year-old G3P2002 at 39-week and 1-day gestation. She noticed reduced fetal movements for which upon examination fetal death was diagnosed. Cervical ripening was started which eventually failed, and surgery was indicated. Findings were an abdominal pregnancy with a third-degree macerated fetus with cord round neck. She was discharged on day 8 postoperation to continue follow-up as an outpatient with regular *β*HCG and ultrasound checks.

**Conclusion:**

This case illustrates the need to effectively confirm an intrauterine location of a pregnancy even in a case of fetal demise and the need to monitor for cord abnormalities in advanced abdominal pregnancy being managed expectantly.

## 1. Introduction

Abdominal pregnancy is a rare form of ectopic pregnancy estimated to occur in 10 out of 100,000 pregnancies in the United States [1]. In recent years, there have been increasing reports of term live abdominal pregnancies. However, preoperative diagnosis is often missed in the majority of cases and is identified in only 45% of cases [2]. Even when diagnosed, the management of advanced abdominal pregnancy is challenging as it is associated with high risk of maternal and perinatal morbidity and mortality. We herein report a case of term abdominal pregnancy with fetal demise due to cord round neck in a resource-limited setting, whilst highlighting the diagnostic challenges.

## 2. Case Presentation

The patient is a 29-year-old G3P2002 woman at 39-week and 1-day gestation, who was referred from a health center for management of fetal demise. She had attended 03 antenatal visits at the same health center wherein the fundal height was documented at each visit and routine care given. Except for occasional painful fetal kicks, she was otherwise well throughout the antenatal period. Three days prior to admission into our service, she experienced reduced fetal movements for which she consulted at the said health center and a diagnosis of fetal demise was made. On admission to our unit, she had stable vital parameters. There was no abdominal tenderness and an apparent fundal height of 32cm was measured, fetal parts were not easily palpable, and fetal heart tones were not perceived. There were no signs of per vaginal bleeding. Her cervix was long, posterior, firm, and closed. An emergency ultrasound scan was requested which confirmed fetal demise (gross cranial deformation was noted) but failed to diagnose an intra-abdominal location.

**Figure 1:**
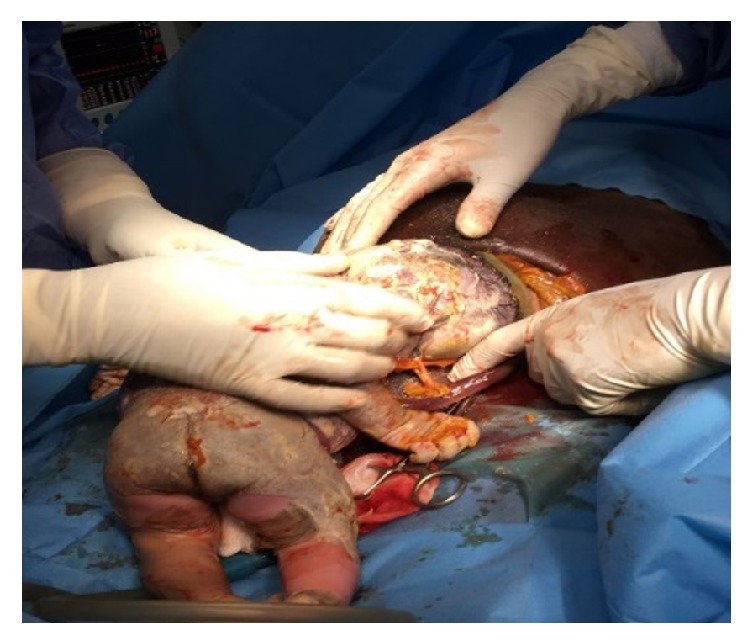
Umbilical cord being removed from the fetal neck.

**Figure 2:**
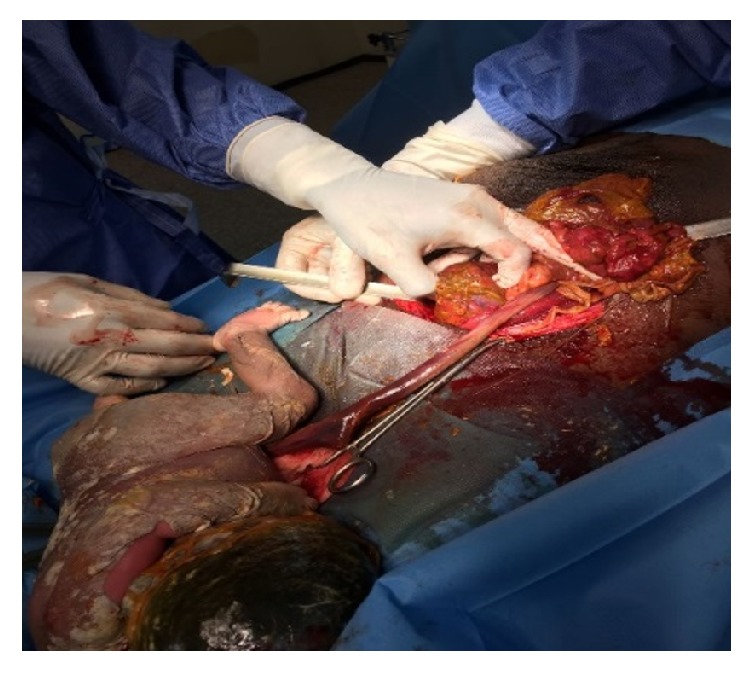
Uterus about 14 weeks.

Cervical ripening was started with 50*µ*g of misoprostol vaginally every 6 hours and after 4 applications; the cervix remained unchanged. A decision for operative delivery was made, indicating failed induction and in a bit to reduce the risk of disseminated intravascular coagulation. The intraoperative findings were a third-degree macerated female fetus floating freely in the peritoneal cavity, weighing 2650g with a tight cord round neck and with no amniotic sac (Figure 1). The uterus was about 14 weeks (Figure 2) and the fallopian tubes and ovaries were normal. The placenta was implanted on the ascending colon and mesocolon. The placenta was left in place. No intraoperative complications were encountered. Postoperatively, she was placed on ampicillin, gentamycin, and metronidazole parenterally for five days. She also received 50mg of methotrexate every other day (04 doses) with 5mg of folinic acid each day proceeding the methotrexate. She was discharged on day 8 postoperation to continue follow-up as an outpatient with regular *β*HCG and ultrasound checks. Her recovery was uneventful with resorption of placenta 6 months later.

## 3. Discussion

Abdominal pregnancy is a rare form of ectopic pregnancy that reaches term. Implantation usually occurs in the peritoneal cavity, outside the fallopian tube and ovary which are commoner extra-uterine sites. Clinical features associated with abdominal pregnancy include persistent abdominal pain, painful fetal movements, palpation of an abdominal mass separate from the uterus, vaginal bleeding, or unaffected and posteriorly displaced pinpoint cervical os amongst others [3]. Ultrasound is an easy and quick method of confirming the gestational location. It is currently the imaging method of choice although, with the presence of gas within the gastrointestinal tract and distorted pelvic anatomy, sonographic interpretation may be difficult especially in advanced cases [4]. Placement of an intrauterine Foley catheter bulb to aid sonographic diagnosis has been described and reported in a case report with successful preoperative diagnosis of advanced abdominal pregnancy [5]. Ultrasound findings of advanced abdominal pregnancy include the absence of a uterine wall surrounding the fetus, fetal parts very close to the abdominal wall, and an abnormal fetal lie [6].

There have been increasing case reports of term abdominal pregnancies with live fetuses. That notwithstanding, abdominal pregnancy carries a higher risk of maternal and perinatal morbidity and mortality. Atrash et al. estimated an associated maternal mortality rate of about 5 per 1000 cases, which is approximately 7 times higher than the estimated rate for ectopic pregnancy in general, and about 90 times the maternal mortality rate associated with normal delivery in the United States [1]. The chances of survival of the newborn is also affected with a perinatal mortality rate of 40% to 95% [7]. In a review of 163 cases [2], Nkusu et al. reported a 72% fetal or perinatal death for advanced abdominal pregnancy.

Our patient had painful fetal kicks, but abnormal palpation of fetal parts was missed during her routine antenatal visits. Even after examination by a doctor in our facility, the fetal parts were not easily palpated. This could be explained in part by the flaccid nature of the decomposing body of the dead fetus. Early diagnosis could reduce the high risk of maternal and perinatal morbidity and mortality associated with abdominal pregnancies. However, it is not uncommon for the diagnosis to be missed, even after evaluation by a specialist as noted in our case. Our patient had a term pregnancy that went up to 39-week gestation. Had the location of the pregnancy been diagnosed earlier, a rigorous monitoring plan could have been implemented and an elective surgery programmed as soon as term is reached. This would have probably given the fetus a favourable outcome. The presence of a tight cord-round-neck most probably explains the cause of death. To our knowledge, cord round neck has not been reported in abdominal pregnancy. This, therefore, suggests that cord abnormalities should be systematically evaluated even in abdominal pregnancies that are being managed expectantly. Perhaps, it is worth reiterating that even when frequent antenatal visits and routine ultrasounds scans are completed, the preoperative diagnosis of abdominal pregnancy is made only in 45% of cases [2]. Therefore, a high index of suspicion is thus required, especially in low- and middle-income countries where resources are often limited [8].

Management of the placenta can be very problematic. Intraoperative removal is the best option; however, it should not be attempted in cases with hemorrhagic risk [9]. Several case reports have been described in which removal of the placenta was associated with massive hemorrhage [2, 6, 10]. Thus unless the placenta blood supply can be easily identified and secured, it is preferable to leave the placenta in situ. Spontaneous resorption of the placenta occurs within several months depending on the individual without which there is increased risk of hemorrhage, sepsis, and necrosis and the patient might need to undergo another surgery [2, 11]. Methotrexate being antimitotic has been used to aid regression of the placenta although more and more authors do not agree with its use. However, whether methotrexate is used or not, follow-up with ultrasound and BHCG should be done. In our case, the placenta was inserted on the ascending colon and mesocolon and was left in place in order to avoid bleeding and extensive excision of viable tissue. We opted for methotrexate administration to aid regression while monitoring for signs of complications. No complications were noted and complete placenta resorption was obtained six months later.

## 4. Conclusion

Abdominal pregnancy is an extremely rare form of ectopic pregnancy associated with high risk of adverse maternal and perinatal outcomes. Our case illustrates the challenges in the timely diagnosis of this condition, thus warranting the need for a high index of suspicion and a thorough clinical and comprehensive ultrasound assessment. Similar to term intrauterine pregnancies, cord round neck poses a risk to fetal well-being, thus, the need to monitor for cord abnormalities in advanced abdominal pregnancies being managed expectantly.

## Data Availability

All data are included within the article.
